# The effect of initial local anesthetic dose with continuous interscalene analgesia on postoperative pain and diaphragmatic function in patients undergoing arthroscopic shoulder surgery: a double-blind, randomized controlled trial

**DOI:** 10.1186/1471-2253-12-6

**Published:** 2012-03-23

**Authors:** Craig T Hartrick, Yeong-Shih Tang, Don Siwek, Robert Murray, David Hunstad, Greg Smith

**Affiliations:** 1 Department of Anesthesiology, Oakland University William Beaumont School of Medicine, Beaumont Hospitals - Royal Oak and Troy, Rochester, MI, USA; 2 Biomedical Sciences and Anesthesiology, Oakland University William Beaumont School of Medicine, 525 O'Dowd Hall, Rochester, MI 48309, USA

**Keywords:** Regional anesthesia, Diaphragmatic paresis, Interscalene block, Shoulder surgery, Dyspnea, Compensatory diaphragmatic function, Randomized controlled trial

## Abstract

**Background:**

Interscalene block (ISB) is commonly performed using 20-40 mL of local anesthetic. Spread to adjacent structures and consequent adverse effects including paralysis of the ipsilateral hemidiaphragm are frequent. Pain ratings, analgesic requirements, adverse events, satisfaction, function and diaphragmatic excursion were compared following interscalene block (ISB) with reduced initial bolus volumes.

**Methods:**

Subjects undergoing arthroscopic rotator cuff repair were randomized to receive 5, 10, or 20 mL ropivacaine 0.75% for ISB in a double-blind fashion (N = 36). Continuous infusion with ropivacaine 0.2% was maintained for 48 h. Pain and diaphragmatic excursion were assessed before block and in the recovery unit.

**Results:**

Pain ratings in the recovery room were generally less than 4 (0-10 NRS) for all treatment groups, but a statistically significant difference was noted between the 5 and 20 mL groups (NRS: 2.67 vs. 0.62 respectively; p = 0.04). Pain ratings and supplemental analgesic use were similar among the groups at 24 h, 48 h and 12 weeks. There were no differences in the quality of block for surgical anesthesia. Dyspnea was significantly greater in the 20 mL group (p = 0.041). Subjects with dyspnea had significant diaphragmatic impairment more frequently (Relative risk: 2.5; 95%CI: 1.3-4.8; p = 0.042). Increased contralateral diaphragmatic motion was measured in 29 of the 36 subjects. Physical shoulder function at 12 weeks improved over baseline in all groups (baseline mean SST: 6.3, SEM: 0.6; 95%CI: 5.1-7.5; 12 week mean SST: 8.2, SEM: 0.46; 95%CI: 7.3-9.2; p = 0.0035).

**Conclusions:**

ISB provided reliable surgical analgesia with 5 mL, 10 mL or 20 mL ropivacaine (0.75%). The 20 mL volume was associated with increased complaints of dyspnea. The 5 mL volume was associated with statistically higher pain scores in the immediate postoperative period. Lower volumes resulted in a reduced incidence of dyspnea compared to 20 mL, however diaphragmatic impairment was not eliminated. Compensatory increases in contralateral diaphragmatic movement may explain tolerance for ipsilateral paresis.

**Trial Registration:**

clinicaltrials.gov. identifier: NCT00672100

## Background

Arthroscopic shoulder surgery is known to be a particularly painful stimulus that historically has often required postoperative hospitalization for intravenous opioid analgesia. Improvements in postoperative analgesia have permitted these procedures to be performed in the ambulatory setting [1]. Consequently, interscalene block (ISB) with continuous interscalene infusion of ropivacaine using pumps has become standard care following shoulder surgery at some centers.

Typically, relatively large boluses of local anesthetic (20-40 mL) are used to initiate the ISB and a continuous infusion of local anesthetic is then added to maintain analgesia. This approach, while effective from an analgesic perspective, is associated with a number of adverse effects. The close proximity of the phrenic nerve, recurrent laryngeal nerve, sympathetic chain, and other portions of the brachial plexus serving the distal extremity predispose patients to transient unwanted diaphragmatic paresis, dysphonia, dysphagia, Horner's syndrome (miosis, ptosis, enophthalmos) with conjunctival injection and nasal congestion, and hand numbness/weakness. While these annoying effects are usually tolerated, they occasionally result in hospitalization for symptom control. Patients with pre-existing pulmonary conditions may not tolerate the sympathectomy or the diaphragmatic paralysis, both of which may occur in more than 80% of subjects [2]. Respiratory compromise or poor pain control are the most common reasons for unscheduled hospitalization. Although reducing the initial local anesthetic bolus from 40 ml to 20 mL in one study still resulted in a 100% incidence of diaphragmatic paralysis [3], decreasing the initial bolus further may result in reduced spread to adjacent neural structures and potentially fewer adverse effects [4]. The reduced mass of local anesthetic also has the added safety benefit of reducing the potential for local anesthetic toxicity.

The objective of this study was to examine the effect of lower initial bolus volumes of local anesthetic on analgesia when using ISB with continuous infusion following arthroscopic shoulder surgery. We hypothesized that lower initial boluses of local anesthetic might result in fewer adverse effects, without adversely impacting the conduct of surgery, additional anesthesia required, or surgical outcome.

## Methods

The specific aims were to: 1) compare pain ratings and supplemental analgesic requirements at discharge from PACU (post anesthesia care unit; recovery room), 24 hours, and 48 hours and 12 weeks following 5, 10, and 20 mL boluses; 2) compare symptomatic adverse events including clinical dysphonia, Horner's syndrome and dyspnea, as well as unexpected hospitalization, evidence of local anesthetic toxicity, and perceived hand weakness at discharge from PACU following 5, 10, and 20 mL boluses; 3) compare impairment in diaphragmatic excursion at discharge from PACU following 5, 10, and 20 mL boluses; 4) compare patients' satisfaction with analgesia at 24 and 48 hours following 5, 10, and 20 mL boluses; 5) compare patient rating of functional outcome at baseline and at 12 weeks following 5, 10, and 20 mL boluses; and 6) compare the rates of general anesthesia required due to inadequate block following 5, 10, and 20 mL boluses.

After IRB approval from the William Beaumont Human Investigation Committee, subjects aged 18-80 years undergoing arthroscopic shoulder surgery were randomized into 3 groups to receive ISB with a 5, 10 or 20 mL initial bolus of ropivacaine 0.75% (http://clinicaltrials.gov. identifier: NCT00672100). The subjects were eligible for enrollment if they were ASA risk class I-III. Exclusion criteria were opioid tolerance, defined as > 40 mg oxycodone equivalent daily over 2 weeks prior to study, significant preexisting pulmonary disease including diaphragmatic paralysis or history of phrenic nerve injury, hypersensitivity to opioids or ropivacaine, known or suspected history of alcohol or drug abuse or dependence within the previous 2 years, history of liver disease, or participation in another clinical study within 30 days of surgery. Written informed consent was obtained from all subjects. Subjects and observers were blinded as to group assignment.

Randomization was provided to the research pharmacist by a biostatistician who used a rotating block technique. The research pharmacist provided the specific patient allocation information directly to the unblinded anesthesiologist immediately prior to the procedure. Unblinded investigators then administered the anesthetic injection and established a continuous infusion through an interscalene catheter in the preoperative holding area. In the operating room the quality of the block was assessed by blinded investigators at the time of surgical manipulation. An inadequate block was defined as either intolerance to preoperative manipulation of the shoulder by the surgeon or failure to demonstrate hypesthesia to pinprick over the C5 and C6 dermatomes as assessed by the blinded anesthesiologist. Sedation with intravenous propofol was then established. Patients with failed blocks and those who were considered intolerant to the positioning (sitting with the head in a restraint device) were provided general anesthesia according to standard practice with propofol for induction, a laryngeal mask airway (LMA) and sevoflurane for anesthetic maintenance. Supplemental postoperative analgesia was provided per patient request in the PACU using intravenous fentanyl (in 25 mcg increments) and intravenous ketorolac (30 mg, one time dose), with oral hydrocodone/acetaminophen (5/500 mg orally every 4 hours as needed) as second-line therapy prior to discharge. A disposable infusion device (Pain Pump II, Stryker, Kalamazoo, MI, USA) was used for continuous infusion with the following settings: ropivacaine 0.2%; bolus: 3 mL; continuous infusion: 4 mL/h; lock-out: 20 minutes. A single dose of dexamethasone was allowed for postoperative nausea and vomiting (PONV) or PONV prophylaxis.

### Interscalene block

Ultrasound-guided placement (high frequency probe; 10-12 Hz) of an ISB and catheter was performed at the ipsilateral upper truck or C5 root, usually near a point midway between lines extending laterally from the cephalad border of the thyroid cartilage and from the cricoid cartilage, between the anterior and middle scalene muscles. Following penetration of the fascial sheath, observation of spread of 1 mL normal saline circumferentially surrounding the most cephalad nerve of the plexus confirmed correct placement in an out-of-plane view. The initial block was placed though the needle (18G Contiplex^®^, B. Braun Medical Inc., Bethlehem, PA, USA) prior to insertion of a non-stimulating 20 gauge nylon catheter. The interscalene catheters were advanced exactly 2 cm in all cases. The catheter used has a closed end with 3 orifices located 5, 10 and 15 mm from the tip. A standardized continuous infusion was started immediately after catheter placement.

### Outcome measures

After discharge home directly from the PACU, patients were contacted by telephone to collect outcome data at 24 hours, 48 hours, and 12 weeks postoperatively using a structured interview.

### Pain measurements

Categorical and Numeric Pain Rating Scales (NRS) were recorded at baseline (pre-operatively), at discharge from PACU, and at 24 and 48 hours. The primary efficacy outcome was the NRS pain score at discharge from PACU. Other pain assessments were secondary endpoints. A change in NRS of 1.8 was considered clinically significant [5].

### Diaphragmatic excursion

Diaphragmatic excursion at maximal effort for inspiration and exhalation was assessed by a blinded ultrasonographer bilaterally, both preoperatively and prior to discharge from PACU, using a low frequency probe (4 Hz) posterolaterally as described by Borgeat et al [6]. A 50% reduction in ipsilateral diaphragmatic motion compared to baseline was considered clinically meaningful and represented the primary safety outcome measure. Because diaphragmatic excursion may decrease as a result of surgery, anesthesia, and opioid pain medications, the change in ipsilateral measurement was also compared to alterations in contralateral diaphragmatic motion.

### Additional secondary outcome measures

#### Dyspnea

At discharge from PACU, 24 and 48 hours patients were asked "Do you feel short of breath or are you having trouble catching your breath?" (yes/no)

#### Subjective dysphonia

At discharge from PACU, 24 and 48 hours patients were asked "Is your voice hoarse?" (yes/no)

#### Symptomatic horner's syndrome

At discharge from PACU, 24 and 48 hours patients were asked "Do you have blurred vision or a droopy eyelid?" If either of these effects were noted the patient was considered to have *symptomatic *Horner's Syndrome and a positive finding (yes/no) was recorded.

#### Perceived hand weakness

At discharge from PACU, 24 and 48 hours grip strength was assessed by asking the question, "Does your grip feel weak?" (yes/no)

#### Patient satisfaction

A 24 and 48 hours the 5-point Likert categorical Helpfulness Scale was administered ("Is your interscalene infusion: extremely harmful; harmful; neutral: not harmful, but not helpful; helpful; extremely helpful?")

#### Functional outcome

At baseline and again at 12 weeks subjects completed the Simple Shoulder Test. This test is a series of 12 (yes/no) questions. This has been shown to be a valid, reliable and consistent for subjects up to and including 60 years of age when similar injuries (rotator cuff dysfunction) are assessed [7]. The use of this assessment collected by telephone interview [8] has been validated and is comparable to more complicated scales when converted to a 100-point scale [9].

#### Local anesthetic toxicity

At discharge from PACU, 24 and 48 hours the presence of any one of the following was considered a positive score: tinnitus, perioral numbness, feeling jittery (yes/no)

#### Other

Unscheduled admission, total amount of local anesthetic used (read directly from the pump), type and amount of supplemental analgesics, nausea/vomiting, requirement for general anesthesia (defined as the use of a laryngeal mask airway and/or inhalational anesthesia, i.e. sevoflurane), early termination (catheter malfunction/dislodgement prior to 48 hours) were recorded.

### Statistical analyses

Sample size for efficacy was determined by considering the equivalence of two means based on means and standard deviations from data previously collected as part of routine quality assurance monitoring and a minimum clinically significant difference for NRS pain at discharge from PACU of 1.8. Twelve subjects per group (N = 36) would yield 90% power at the 0.05 significance level. Power analysis for primary safety outcome (proportion of subjects with significant reductions in diaphragmatic excursion) using a 30% difference from expected outcome (5 mL vs. 20 mL) with 12 subjects in each group provided 90% power at the 0.01 significance level.

For each bolus group, most outcomes were measured repeatedly over time. Therefore, measurements obtained from a patient in each group over time were not independent. Continuous outcomes measured at only one time point were compared across the three bolus groups using either a one-way analysis of variance or a Kruskal-Wallis test depending on whether the response is normally or non-normally distributed respectively. Categorical outcomes were compared among the three bolus groups using either the Fisher's Exact or Chi-Square tests. Continuous outcomes measured at exactly two time-points were analyzed by first computing the paired differences (accounting for correlation in measurements between time points) within each bolus group and then analyzing the differences across the three bolus groups using a one-way analysis of variance or a Kruskal-Wallis test depending on whether the paired differences is normally or non-normally distributed respectively. Categorical outcomes were analyzed using the Cochran-Mantel-Haenszel statistics stratified by the bolus groups.

Continuous and categorical outcomes measured at more than two time-points were analyzed in the following manner: Normally and non-normally distributed outcomes measured were analyzed using generalized linear mixed models (GLMM), nonlinear mixed models (NLMM), general linear models (GLM), or nonlinear models (NLM) depending on whether the errors are correlated and/or the presence of random effects, and/or the presence of nonlinearity. To make the analysis more sensitive to individual variations, baseline outcome measurements were used as covariates in these analyses thereby altering all of these models to analyses of covariance (ANCOVA). All of the data were analyzed on an intent-to-treat principle using SAS JMP 8.0.

Additionally, all outcome measures were first analyzed using numerical and graphical techniques to determine their distributions. Based on this preliminary assessment, parametric, nonparametric, or exact statistical tests were used. Functional outcomes based on the Simple Shoulder Test were first scored according to the previously mentioned algorithm [9]. Any subscales generated based on this algorithm were then be used in the analysis instead of the individual items.

## Results

**Figure 1:**
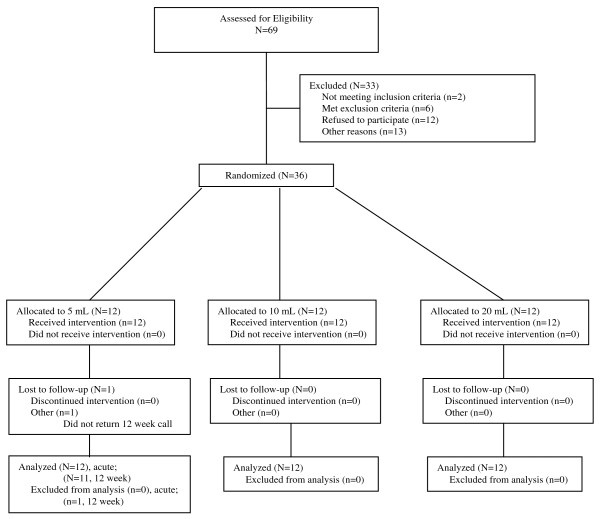
**CONSORT diagram**.

Sixty-nine subjects were screened; from January 2009 through December 2009, 36 subjects were randomized into the study (Figure 1). All subjects completed the study with the exception of one subject in the 5 mL group who was lost to follow-up for the 12-week endpoint only. All patients were managed as outpatients; none were admitted to hospital and no patient experienced a prolonged recovery stay. Demographics and baseline pain scores were similar among all three groups (Table 1).

**Table 1:** Demographics and Baseline Evaluations

	5 mL (N = 12)	10 mL (N = 12)	20 mL (N = 12)	P value
Age (mean in yr ± SEM; 95%CI)	51 ± 4; 42-60	55 ± 3.7; 46-63	50.5 ± 3.3; 43-58	> 0.72

Sex (M/F)	7/5	9/3	9/3	> 0.78

BMI(mean in kg/m^2 ^± SEM; 95%CI)	29 ± 2; 25-33.4	28 ± 0.9; 26-30	29 ± 1.9; 25-33	> 0.82

ASA status (1/2/3)	2/8/2	0/12/0	3/8/1	> 0.512

Baseline Pain (median NRS: 0-10; 95%CI)	2; 0.7-4.5	2; 0.6-2.6	2; 0.8-2.3	> 0.52

Dominant hand (R/L)	12/0	10/2	11/1	> 0.22

Operative side (R/L)	9/3	7/5	7/5	> 0.52

Baseline Function (SST median; 95%CI)	7; 4.5-9.5	5; 3.5-7.8	7; 4.2-8.4	> 0.50

Baseline diaphragmatic excursion: right (mean ± SEM; 95%CI)	4.5 ± 0.7; 3.0-6.0	4.5 ± 0.6; 3.1-5.9	4.6 ± 0.7; 3.1-6.1	> 0.90

Baseline diaphragmatic excursion: left (mean ± SEM; 95%CI)	4.1 ± 0.5; 3.0-5.2	4.5 ± 0.5; 3.4-5.7	4.2 ± 0.4; 3.3-5.1	> 0.81

SEM: Standard Error of the Mean; 95%CI: 95% Confidence Interval; BMI: Body Mass Index; ASA: American Society of Anesthesiologists; NRS: numeric rating scale; SST: Simple Shoulder Test

Pain ratings and supplemental analgesic use were generally similar among the groups at 24 h, 48 h and 12 weeks (Table 2). While the mean pain scores at all time points and for all groups were less than 4 (0-10; NRS), a statistically significant difference in pain at discharge was noted when comparing the 5 mL group to the 20 mL group (NRS: 2.67 vs. 0.62 respectively; p = 0.04). Supplemental opioid was numerically greater in the first 24 hours in the 5 and 10 mL groups compared to the 20 mL group, but this difference was not statistically significant (Table 3). The use of the patient-controlled regional analgesia, as measured by the total ropivacaine administered via the pump, was also similar among all 3 groups over the first 48 hours.

**Table 2:** Postoperative Pain [mean NRS (SE); 95%CI]

Allocation	Discharge	24 h	48 h	12 weeks
5 mL	2.67 (0.93); 0.61-4.71*	3.67 (0.74); 2.03-5.30	2.67 (0.72); 1.08-4.25	1.5 (0.45); 0.50-2.50

10 mL	1.58 (0.80); -0.18-3.35	3.33 (0.80); 1.57-5.10	2.08 (0.65); 0.66-3.50	2.42 (0.85); 0.55-4.28

20 mL	0.62 (0.40); -0.26-1.49*	2.54 (0.67); 1.09-3.99	2.15 (0.56); 0.92-3.38	1.15 (0.34); 0.42-1.89

NRS: numeric rating scale; SE: Standard Error; 95%CI: 95% Confidence Interval * p = 0.04 for 5 mL compared to 20 mL only; for all other comparisons p > 0.05

**Table 3:** Postoperative analgesics [mean (SE); 95%CI]**

Allocation	opioid 0-24 h* (mg)	opioid 24-48 h* (mg)	cumulative ropivacaine infusion 0-24 h (mL)	cumulative ropivacaine infusion 0-48 h (mL)
5 mL	27.7 (8.4); 8.9-46.5	18.9 (6.2); 3.7-34.1	111.3 (8.0); 93.7-128.9	210.8 (16.9); 173.6-248.0

10 mL	29.8 (8.2); 11.9-47.7	16.4 (3.5); 8.4-24.4	131.6 (12.4); 104.4-158.8	236.1 (9.9); 214.3-257.9

20 mL	13.4 (1.7); 9.6-17.3	21.3 (4.1); 12.3-30.3	113.0 (4.9); 102.4-123.6	219.8 (10.9); 196.0-243.5

SE: standard error; 95%CI: 95% confidence interval

* converted to equivalent oral morphine (mg)

**p > 0.05 for all comparisons

There were no differences in the quality of block for surgical anesthesia. This determination was made by the blinded anesthesiologist prior to surgery. Allocation had no effect on type of anesthesia used: the majority had sedation only (n = 15) or GA per surgeon request for positioning (n = 17). Inadequate analgesia, as determined by the blinded anesthesiologist prior to surgery, requiring GA was not significantly different among groups (5 mL: n = 1; 10 mL: n = 2; 20 mL: n = 1). No patient was converted from sedation to GA.

The subjective sensation of dyspnea in the PACU was significantly greater in the 20 mL group (5 mL: 1 (8.3%); 10 mL: 0 (0%); 20 mL 4 (33%); Likelihood ratio 6.4, p = 0.041). While mean reductions in ipsilateral diaphragm motion were noted following interscalene block in all groups (Table 4), independent of dose, subjects with dyspnea frequently exhibited clinically meaningful ipsilateral diaphragmatic impairment (Relative risk: 2.5; 95%CI: 1.3-4.8; p = 0.042). All subjects reporting dyspnea had Body Mass Indices greater than 30. The subject with dyspnea in the 5 mL group had a BMI of 31; the subjects in the 20 mL group had BMIs of 31, 32, 33, and 33. However, there was no direct correlation generally between BMI and dyspnea. Moreover, there was an increase in contralateral diaphragmatic excursion noted in 29 of the 36 subjects (Table 4). Analysis of covariates failed to find a clinically meaningful correlation between the degree of ipsilateral reduction in hemidiaphragm excursion and the increase in contralateral function. None of the patients experienced significant oxygen desaturation requiring intervention or resulting in a prolonged recovery time. Other adverse effects were also similar among groups. There were no unscheduled admissions or early terminations.

**Table 4:** Diaphragmatic Change

	5 mL (N = 12)	10 mL (N = 12)	20 mL (N = 12)	P value (between groups)
reduction ipsilateral (% ± SEM; 95%CI)	66 ± 10; 43 to 88	60 ± 17; 21-98	65 ± 9; 45-86	p > 0.05
reduction contralateral (% ± SEM; 95%CI)	-28 ± 7.7; -45 to -10	-56 ± 38; -139 to 26	-39 ± 33; -114 to 36	p > 0.05

SEM: Standard error of the mean; 95%CI: 95% confidence interval

Patient satisfaction was high in all groups at both 24 and 48 hours. The majority of subjects considered the analgesic technique to be either "helpful" or "extremely helpful" at 24 and 48 hours respectively in the 5 mL group (83%; 83%), the 10 mL group (83%; 75%), and the 20 mL group (92%; 85%). There were no statistically significant differences in satisfaction among groups. Surgical outcome, as assessed by the SST, improved similarly and significantly in all groups (baseline mean SST: 6.3, SEM: 0.6; 95%CI: 5.1-7.5; 12 week mean SST: 8.2, SEM: 0.46; 95%CI: 7.3-9.2; p = 0.0035).

## Discussion

Interscalene block provides an effective method for both surgical anesthesia and postoperative analgesia in shoulder surgery. While injection of relatively large volumes of local anesthetic may increase the duration of anesthesia, perhaps by providing a reservoir effect, spread of local anesthetic away from the plexus (as occurs with a large bolus of medication injected over a relatively short period of time) results in unwanted blockade of other neural structures in the cervical region, including the phrenic nerve or its constituent nerve roots. In a study by Riazi et al. examining diaphragmatic function following interscalene block comparing 5 mL to 20 mL injection of ropivacaine 0.5%, significantly fewer subjects experienced diaphragmatic paresis in the lower volume group [10]. This sparing effect was accompanied by a blunting of impairments in FVC and oxygen saturation without a reduction in postoperative analgesia. However, lower amounts of local anesthetic (reduced mass of drug), while satisfactory for postoperative analgesia, could potentially result in diminished effectiveness of the quality of surgical anesthesia. Since all subjects were provided general anesthesia for their operative procedure, whether the low volume injections would have been adequate for surgery is unknown.

A recent report comparing ISB at the C6 level with 10 mL to 20 mL also found no difference in postoperative analgesia duration or quality [11]. However, as in the Riazi et al. study, all subjects were given a general anesthetic, including neuromuscular blockade. In contrast to Riazi et al., injection of the lower volume (10 mL as opposed to 5 mL) did not result in sparing of diaphragmatic function. In the present study, when ISB was performed with US-guidance directed at the C5 level, reliable surgical anesthesia was achieved with 5 mL ropivacaine (0.75%) for shoulder arthroscopy for rotator cuff repair. The requirement for general anesthesia due to inadequate blockade was low and similar in all groups. Even though the assessment as to the quality of block was made by a blinded anesthesiologist before sedation was given, the fact that GA was allowed as an option is a limitation of the study. No subjects were converted from sedation to GA and no patients required neuromuscular blockade. The use of a higher concentration of local anesthetic (0.75% vs. 0.5%) in this study may have contributed to the quality of block for surgical anesthesia.

Although the pain scores on discharge from the recovery room were numerically higher in the 5 mL group compared to the 20 mL group, clinically all pain scores remained low and were well tolerated, perhaps as a result of the small statistically non-significant increase in the supplemental opioid in both the 5 mL and 10 mL groups compared to the 20 mL group over the first 24 hours. Others have reported satisfactory postoperative analgesia with volumes even lower than 5 mL. McNaught et al. reported that the use of ultrasound for interscalene block allowed the mean effective volume of local anesthetic for adequate postoperative analgesia in the immediate recovery period to be reduced to 0.9 mL compared to 5.4 mL when using nerve stimulation [12]. Similar findings were reported by Renes et al. where the minimum effective volume of 0.75% ropivacaine was 2.9 ml in 50% of patients and 3.6 mL in 95% of patients [13]. The postoperative analgesia with the 5 mL volume for interscalene block in the present study was less impressive; the difference in mean NRS in the PACU between the 5 mL and 20 mL groups was greater than the prespecified 1.8 point differential defined as a clinically significant change in pain scores. However, with mean NRS scores for pain less than 3, it did not translate into delayed discharge, lower satisfaction, or statistically higher supplemental opioid. Clinically the quality of postoperative analgesia could potentially be improved by repeating a small volume injection through the interscalene catheter at the conclusion of surgery or in the recovery room without risking the widespread distribution associated with a large initial bolus. Despite these differences, patient satisfaction with the quality of postoperative analgesia was high and similar in all groups.

While no subjects required admission to hospital, the 20 mL volume was associated with increased subjective complaints of dyspnea consistent with a previous report [10]. However, in contrast to their results [10] and the results of Renes et al. [13], the use of lower volume (5 mL) did not result in consistent sparing of diaphragmatic function. Whether this was the result of the use of a higher concentration (0.75% versus 0.5%) [10] and thus a larger mass of drug, or the precise placement at C5, as opposed to a lower placement adjacent to the C6 [10] or C7 [13] cannot be determined from this study. It is likely that even small precisely placed volumes of local anesthetic, when placed at C5, can spread proximally to affect the phrenic nerve (C4,5) function even when there is no spread anterior to the anterior interscalene muscle. Spread to the sympathetic chain with subsequent Horner's Syndrome was evaluated only on a symptomatic basis. Larger initial injectate volumes, while inducing more objective evidence of Horner's Syndrome preoperatively, did not result in increased subjective complaints in the postoperative period. This effect was therefore considered well tolerated in patients without preexisting respiratory compromise or asthma.

A compensatory increase in contralateral diaphragmatic movement could account for the relative tolerance for unilateral paresis in otherwise healthy subjects without preexisting respiratory disease. However, the degree of compensation did not correlate well with the degree of ipsilateral paresis. This suggests the involvement of other covariants. All patients in this study with subjective complaints of dyspnea were obese. It seems reasonable that an increased BMI could play a role in the reduced ability to effectively increase the excursion of the contralateral hemidiaphragm. Yet a clear inverse relationship between BMI and contralateral diaphragmatic compensation was not observed. Consequently, while obesity could be one risk factor, since other obese subjects did not develop dyspnea, it seems not to be sufficient as the sole risk factor. The study was not sufficiently powered to further evaluate this effect.

## Conclusions

As the highest dose was associated with increased dyspnea, absent improvement in surgical anesthesia or significantly improved subsequent analgesia, function, or satisfaction scores, the 20 mL initial bolus cannot be routinely recommended. This recommendation may be especially valid when an interscalene catheter is placed, thus allowing additional incremental local anesthetic injections to be titrated to control postoperative pain. While the 5 mL initial bolus generally provided adequate analgesia in the PACU, it was associated with statistically higher pain scores and a trend towards greater opioid consumption compared to the 20 mL group. This suggests that the 5 mL group, having no advantage over the 10 mL group with respect to reduced adverse effects, might be near the lower limit of clinical acceptability. Lower concentrations of local anesthetic might further reduce complaints of dyspnea, however the effect on the quality of surgical anesthesia when using a lower mass of drug has not been evaluated.

## Competing interests

Dr. Siwek is a consultant for Stryker Corporation. All other authors declare they have no competing interests.

## Authors' contributions

CH and DS conceived the study. CH and YT designed the study. DH was responsible for standardization of the injection technique; YT and RM were responsible for standardization of the diaphragmatic assessment technique. All authors participated in study design and the conduct of study. CH and YT were responsible for data analysis and manuscript preparation. All authors reviewed and approved the final manuscript.

## Pre-publication history

The pre-publication history for this paper can be accessed here:

http://www.biomedcentral.com/1471-2253/12/6/prepub
